# The effects of Borrelia infection on its wintering rodent host

**DOI:** 10.1007/s00442-022-05272-y

**Published:** 2022-10-15

**Authors:** Saana Sipari, Jukka Hytönen, Annukka Pietikäinen, Tapio Mappes, Eva R. Kallio

**Affiliations:** 1 Department of Biological and Environmental Science, University of Jyväskylä, PO Box 35, 40014 Jyväskylä, Finland; 2 Institute of Biomedicine, University of Turku, Kiinamyllynkatu 10, 20520 Turku, Finland; 3 Clinical Microbiology, Tyks Laboratories, Turku University Hospital, Kiinamyllynkatu 10, 20520 Turku, Finland

**Keywords:** *Borrelia afzelii*, *Myodes glareolus*, Autumn–winter, Host–pathogen interactions, Zoonosis

## Abstract

In seasonal environments, appropriate adaptations are crucial for organisms to maximize their fitness. For instance, in many species, the immune function has been noticed to decrease during winter, which is assumed to be an adaptation to the season’s limited food availability. Consequences of an infection on the health and survival of the host organism could thus be more severe in winter than in summer. Here, we experimentally investigated the effect of a zoonotic, endemic pathogen, *Borrelia afzelii* infection on the survival and body condition in its host, the bank vole (*Myodes glareolus*), during late autumn–early winter under semi-natural field conditions in 11 large outdoor enclosures. To test the interaction of *Borrelia* infection and energetic condition, four populations received supplementary nutrition, while remaining seven populations exploited only natural food sources. Supplementary food during winter increased the body mass independent of the infection status, however, *Borrelia afzelii* infection did not cause severe increase in the host mortality or affect the host body condition in the late autumn–early winter. While our study suggests that no severe effects are caused by *B. afzelii* infection on bank vole, further studies are warranted to identify any potentially smaller effects the pathogen may cause on the host fitness over the period of whole winter.

**Supplementary Information:**

The online version contains supplementary material available at 10.1007/s00442-022-05272-y.

## Introduction

Seasonality plays a major role in the life history of many organisms (Stearns 1992). Seasonally appropriate behaviour and adaptations are crucial for organisms to maximize their fitness (Boyce 1979; Conover 1992; Kozlowski 2006; Varpe 2017). Strongest seasonality occurs in high latitude areas, like the Arctic and boreal regions, where breeding season often lasts for short periods and takes place during spring and summer, when food is abundant and temperatures are favourable, whereas winters are characteristically cold and long, with limited energy supply (Marchand 1996). For winter-active species, surviving the winter with limited energy resources combined with elevated investment on thermoregulation requires allocating resources from less immediately vital functions (Lochmiller and Deerenberg 2000; McEwen and Wingfield 2003; McNamara and Buchanan 2005; Beldomenico et al. 2008a; Bronson 2009). For instance, in many species, the immune function has been noticed to decrease during winter, which is assumed to be an adaptation to the season’s limited food availability (Svensson et al. 1998; Lochmiller and Deerenberg 2000; Møller et al. 2003; Beldomenico et al. 2008b). The risk of many pathogen infections is generally lower during winter, decreasing the need for a strong immune response (Beldomenico et al. 2008b), however, due to the impaired immune function, combined with the limited energy resources and increased energy demands, the consequences of an infection on the health and survival of the host organism could be more severe in winter than in summer.

Pathogens are suggested to play an important role in regulating population dynamics (Tompkins and Begon 1999; Tompkins et al. 2001), yet, the role of winter in disease ecology has gained fairly little attention, despite the winter-time impairment of immune function reported in many species (Svensson et al. 1998; Lochmiller and Deerenberg 2000; Møller et al. 2003; Beldomenico et al. 2008b). Pathogens can have a direct effect on overwintering survival, thus, partly determining the size of the breeding population in the following spring (Telfer et al. 2002; Kallio et al. 2007; Pedersen and Greives 2008; Kloch et al. 2013). In a winter-active small mammal, the bank vole (*Myodes glareolus*), an endemic viral infection caused by the Puumala orthohantavirus (PUUV) causes decreased winter survival, whereas in summer, the infection has no effect, or even seems to increase the vole survival (Kallio et al. 2007; Tersago et al. 2012; Reil et al. 2017). Similar pattern was observed with a cowpox virus infection in bank voles and wood mice (Telfer et al. 2002). Apart from the aforementioned examples, the winter-time pathogen–host interactions and their potential effects on the overwintering hosts are largely unknown.

In this study, we tested the effect of an endemic *Borrelia afzelii* infection on its wintering host, the bank vole. A spirochaete bacterium *B. afzelii* (*B. burgdorferi* sensu lato complex) is one of the causative agents of human Lyme borreliosis, the most common vector-borne disease in Europe (Kurtenbach et al. 2006; Rizzoli et al. 2011; Stanek et al. 2012). The pathogen is transmitted by *Ixodes* ticks, while rodents are the main reservoir host of the pathogen (Hanincova et al. 2003; Mannelli et al. 2012). In humans, Lyme borreliosis causes serious morbidity, including skin, joint and nervous system manifestations, and can lead to chronic illness if left untreated (Stanek et al. 2012). In animal hosts, the symptoms of chronic *B. burgdorferi* s. l. infections and their long-term effects are less evident and especially under field conditions relatively unknown (Yrjänäinen et al. 2006; Schwanz et al. 2011; Voordouw et al. 2015; Salo et al. 2015; Ostfeld et al. 2018; Cayol et al. 2018). In studies of white-footed mice (*Peromyscus leucopus*), no effect on survival or activity was observed (Schwanz et al. 2011; Voordouw et al. 2015; Ostfeld et al. 2018). Furthermore, in red-backed voles (*Myodes gapperi*), an artificial infection with *B. burgdorferi* caused no clinical disease in the test animals (Bey et al. 1995). In the study of Cayol et al. (Cayol et al. 2018), an artificial *B. afzelii* infection in bank voles had no effect on survival, but affected the fitness via sex and density specific effects on reproduction in summer season. However, the effect of *B. afzelii* infection on its wintering rodent host has never been tested.

The aim of our study was to quantify whether experimental *B. afzelii* injection causes strong reduction on the survival and/or body condition in wintering bank voles. To test for the interaction of energetic condition and *Borrelia* infection on wintering bank voles, we further executed a food manipulation. We monitored the bank voles in large outdoor enclosures successfully from October to December. We hypothesized that *Borrelia* injected animals receiving no food supplementation would express lower body condition and survival compared to other groups. Uninfected individuals with supplementary food were expected to survive best during the winter, and express good body condition.

## Materials and methods

### Experimental design

#### Test animals

The experimental animals were descendants of wild caught individuals, mainly first and second generation. We used 88 laboratory born bank voles (44 females and 44 males), born in July–August 2020. The animals were maintained in the animal facility at the University of Jyväskylä (for more details, see (Cayol et al. 2018) “supplementary material”). Half of the animals were injected with *B. afzelii* (*Borrelia* treatment, BT), whereas the control animals received saline injections (control treatment CT). The sample size was estimated to be sufficient to detect 50% (or 75%) decrease in survival in BT individuals in comparison to CT individuals, which survival was expected to be 60% in December (or 35% in March) (Sipari et al. 2016; van Cann et al. 2019) The estimation was calculated using the power analyses calculator by Kane 2019. The expected effect was similar to the decrease in winter survival caused by PUUV in bank vole (Kallio et al. 2007).

#### Manipulations

BT individuals were injected with a dose of 10^5^ bacteria of a local strain of *B. afzelii* (Cayol et al. 2018), suspended in 0.1 ml of phosphate buffered saline (PBS). CT individuals received PBS (0.1 ml). All injection were given subcutaneously. The manipulations took place in early October 2020, after which the study individuals spent 3 weeks in a non-heated green house to acclimatize to natural day length (October) and outdoor temperatures. The animals were housed in standard mouse cages (43 × 26 × 15 cm; Tecniplast, Italy) with wood shavings and hay for beddings. Water and food (standard rodent pellets, Avelsfoder för råtta och mus R36; Lactamin, Stockholm, Sweden; 18.5% protein; 4.0% fat; 55.7% carbohydrate, 1260 kJ metabolizable energy/100 g) were provided ad libitum*.*

After the acclimation period, all bank voles were released into 11 outdoor enclosures (each 0.2 ha), situated in the vicinity of Konnevesi Research Station (see (Cayol et al. 2018) “supplementary material”), in late October. At the time of release, the numbers of animals available to the treatment and control groups were 44 females (22 infected, 22 uninfected) and 43 males (23 infected, 20 uninfected). In each of the enclosures, we released eight individuals (two BT females, two BT males, two CT females, two CT males), except in one enclosure only seven individuals (see details in Table S1). The enclosures were further distributed into groups receiving supplementary food (four enclosures with supplementary food, seven enclosures without food: Table S1). Individuals were randomly distributed into the treatment groups, and there were no significant differences in the body mass between different groups in the beginning of the experiment (*T* test: Food treatment; *t* = − 1.109, df = 64.381, *p* value = 0.271, *Borrelia* treatment; *t* = 0.243 df = 81.186, *p* value = 0.809). Supplementary food was the same the animals consume in the animal facilities. The food was provided in eight own made feeding stations in weather protected chimneys in the enclosure. The enclosures were visited frequently (every second week until January, after that once per month) to ensure ad libitum food throughout the study in the four food supplement enclosures.

#### Trapping and sampling

The bank voles were captured for the first time in mid-December, using Ugglan live traps. In each enclosure, 20 traps are permanently held in galvanised metal chimneys, where the trap (and feeding station) is protected from weather. Voles can enter the chimney through the open bottom and the trap can be accessed through a lid. The feeding stations were removed and the traps were prebaited with sunflower seeds for 3 days before the traps were baited for the actual trapping with sunflower seeds, piece of potato and filled with bedding material (wood wool for pets). The traps were checked 2 times per day and the trapping lasted for 3 days. All enclosures were trapped simultaneously. The second survival trapping session was carried out in early March in the same way as in December. However, due to drastically decreased survival later in the winter, the second session (March) was excluded from the data analyses.

Captured individuals were taken to the laboratory for body condition measurements (body mass, head width, signs of reproduction) and sampling. Blood sample (< 90 µl) was taken from the retro-orbital sinus with capillary tubes (Haematocrit capillaries, Hirschman Laborgeräte, Germany) and placed in plasma tubes, centrifuged (6000 rpm for 10 min) and stored at − 20 °C until use. Body condition index was calculated by regressing the body mass on head width, and using the residuals as the index value (Oksanen et al. 2002). Reproductive status (mature/immature) was determined based on the signs of breeding (vaginal opening and nipples in females, scrotal testes in males). All captured voles were returned to their original enclosures within 2 days.

#### Laboratory analyses

The comparisons between BT and CT individuals were done based on the given treatment as no blood sample was taken before the animals were released into the enclosures. The plasma samples taken in mid-December were used to an ELISA assay to detect IgG antibodies against *B. afzelii* as described (Cayol et al. 2018). Briefly, Microtiter plates (Thermo Fisher Scientific) were coated with *B. afzelii* cell lysate diluted in PBS (concentration 10 μg/ml) at 37 °C overnight and washed three times with Aqua-Tween (H2O, 0.05% Tween 20, Merck). The vole serum samples were diluted 1:100 in PBS with 1% bovine serum albumin (BSA, Serological Proteins Inc., Kankakee, USA). The samples were incubated on the plates at 37 °C for 1 h, washed three times, and incubated with HRP-conjugated goat anti-mouse IgG secondary antibody (final concentration 0.08 μg/ml, Pierce, Thermo Fisher Scientific) in PBS at 37 °C for 1 h. After the last three washes, ortho-phenylene-diamine (OPD, Kem- En-Tec Diagnostics A/S, Uppsala Sweden) substrate was added for 15 min before the reaction was stopped with 0.5 M H2SO4. The absorbance was measured at a wavelength of 492 nm with Multiskan EX spectro-photometer (Thermo Fisher Scientific). A positive control (serum from laboratory mouse infected with *B. burgdorferi* sensu stricto as confirmed by culture, a negative control (serum from uninfected mouse) and blank without sample were included in each plate. All samples were run in duplicate. The cut-off value for positive result was estimated based on the absorbance of CT individuals (= mean absorbance + 3*standard deviation). Cut-off absorbance for positive result was 0.111.

#### Statistical analyses

To test whether *Borrelia* treatment affected the bank voles’ probability of survival or being reproductively active at the first trapping (mid-December), we used generalized linear mixed models (GLMM, glmer function in r package lme4 (Bates et al. 2015)) with binomial response variable (alive/dead or immature/mature). In the survival analysis, we used *Borrelia* treatment (injected vs. control), food treatment (supplementary food vs no supplementary food), sex, and the initial bodymass of the individual (measured in early October, before the injections),and their relevant interactions as fixed factors, and enclosure ID as a random factor. The reproductive status analysis was carried out with the same set of variables as for survival analysis, but due to the low sample size and unevenly distributed cases between the treatment groups, no tests for interactions were feasible. Antibody level, body condition index and body mass were tested using linear mixed model (LMM, lme function in nlme package (Pinheiro et al. 2007)), with *Borrelia* treatment, food treatment, sex, initial body mass and their interactions as fixed factors and enclosure ID as random factor. Model selections were carried out based on Akaike information criteria for small sample size (AICc) using function dredge (MuMIn package (Barton 2009)). For reproductive status, model selection is not reported, as the full model was used for the final analysis. The variables *Borrelia* treatment, food treatment, sex and initial body mass were included in all considered models. The simplest model within two units of the smallest AICc value was selected as the best model, and the best models are provided in the results. The model selection tables are presented in Online Resource 1, Supplementary Table S2. To ensure the random distribution of individuals between the treatment groups in the beginning of the experiment, the initial body mass was tested with t test for food treatment and *Borrelia* treatment (Table 2). To test for the seroconversion success of the BT individuals, we used Fisher’s exact test (Figure S1).

During the experiment, in total five individuals had been able to escape their original enclosure to an adjacent enclosure. Two individuals from a food supplemented enclosure into another food supplemented enclosure, and three individuals from non-food supplemented enclosure into a supplemented enclosure. The three individuals who switched from a non-food supplemented enclosure into a supplemented enclosure, have been excluded from the GLMM and LMM analyses. All the analyses were carried out using R, version 4.1.3 (R Core Team 2022).

## Results

Of the 87 individuals released in the enclosures, 45 were recovered in the first trapping session (mid-December). The survival rate was 60% among BT individuals and 43% among CT individuals. Neither *Borrelia* treatment, food treatment, sex, nor the initial body mass had effect on survival (Table 1). In the second survival trapping session in early March, only three individuals were recovered (overall survival rate 3%). No measurements were taken due to the insufficient sample size.

**Table 1:** The effect of *Borrelia* treatment, food treatment, sex and initial body mass* on A. bank vole survival till mid-December and B. signs of breeding condition in mid-December

A. Survival				
Explanatory variable	Estimate	Std. error	*z* value	*p* value
Intercept**	0.471	1.676	0.281	0.779
*Borrelia* treatment	0.783	0.504	1.553	0.120
Food treatment	0.561	0.767	0.731	0.465
Sex	0.436	0.551	0.792	0.429
Initial body mass	− 0.039	0.087	− 0.458	0.647

B. Signs of reproduction				
Explanatory variable	Estimate	Std. error	*z* value	*p* value
Intercept	− 8.010	6.970	− 1.149	0.250
*Borrelia* treatment	− 0.030	1.383	− 0.021	0.983
Food treatment	6.101	4.145	1.472	0.141
Sex	2.017	2.465	0.818	0.413
Initial body mass	0.372	0.362	1.027	0.304

*Initial body mass did not differ significantly between treatments in the beginning of the experiment (t test: Food treatment; p value = 0.271, Borrelia treatment; p value = 0.809), **Intercept represents a control female released into enclosure without supplementary food

*Borrelia* treatment or sex had no effect on bank vole body mass, body condition index or signs of reproduction in December (Tables 1, 2). Food treatment and initial body mass had significant effect on body mass but not on body condition index nor reproductive status (Tables 1, 2). Signs of reproductive activity were observed in 13 individuals, of which 11 were receiving supplementary nutrition, however, the effect of food did not reach significance in our model (Table 1).

**Table 2:** The effect of *Borrelia* treatment, food treatment, sex and initial body mass on A. bank vole body mass, B. body condition index and C. antibody absorbance levels in mid-December

A. Body mass					
Explanatory variable	Estimate	Std. error	df	*t* value	*p* value
Intercept	15.763	2.674	25	5.894	< 0.001
*Borrelia* treatment	− 0.501	0.749	25	− 0.669	0.510
Food treatment	2.274	0.842	9	2.701	0.024
Sex	− 0.543	0.857	25	− 0.633	0.532
Initial body mass	0.302	0.144	25	2.099	0.046

B. Body condition index					
Explanatory variable	Estimate	Std. error	df	*t* value	*p* value
Intercept	1.010	1.154	25	0.875	0.390
*Borrelia* treatment	− 0.107	0.322	25	− 0.331	0.744
Food treatment	0.613	0.371	9	1.651	0.133
Sex	0.099	0.370	25	0.268	0.791
Initial body mass	− 0.035	0.062	25	− 0.559	0.581

C. Antibody levels					
Explanatory variable	Estimate	Std. error	df	*t* value	*p* value
Intercept	0.094	0.023	24	4.035	< 0.001
*Borrelia* treatment	0.063	0.020	24	3.16	0.004
Food treatment	− 0.005	0.020	9	− 0.249	0.809
Sex	− 0.007	0.019	24	− 0.360	0.722
Initial body mass	0.088	0.074	21	1.186	0.249

Intercept represents a control female released into enclosure without supplementary food

Antibodies against *Borrelia* infection were measured from 40 individuals that were sampled for blood in mid-December. *Borrelia* treatment had a significant effect on antibody levels (Table 2). Compared to control individuals, BT individuals expressed significantly higher antibody levels (Figure S1). Out of the 27 BT individuals sampled in mid-December, 13 were seropositive, showing that there is a significant association between the *Borrelia* treatment and the likelihood of being seropositive (Fisher’s exact test *p* = 0.003). There were no seropositive individuals in the CT group.

## Discussion

In this study, we experimentally tested the effect of *B. afzelii* infection on the survival and body condition in its wintering host, the bank vole. To our knowledge, this experiment is the first to examine the role of winter in the pathogen–host interactions of *B. burgdorferi* sensu lato in its natural host. Our results indicate that *B. afzelii* infection does not increase mortality in bank voles during late autumn–early winter. Furthermore, we found the infection to have no effect on the body condition of wintering individuals. However, supplementary food during early winter increased the body mass and induced winter breeding (albeit not quite significantly) in bank voles independent of their infection status.

Our results are in line with earlier findings, suggesting that *B. burgdorferi* sensu lato does not affect the survival of its reservoir hosts (Schwanz et al. 2011; Voordouw et al. 2015; Ostfeld et al. 2018; Cayol et al. 2018). However, earlier studies investigating the effect of *Borrelia* infection on its host’s survival, behaviour or physiology, are mainly performed during the breeding season or under laboratory conditions (Yrjänäinen et al. 2006; Schwanz et al. 2011; Voordouw et al. 2015; Salo et al. 2015; Ostfeld et al. 2018; Cayol et al. 2018). In northern high latitude areas, winter conditions are often extreme, exposing organisms to short daylengths, cold temperatures and limited food sources for a long period of time. Short day length is known to affect multilevel endocrine functions in mammals (Moffatt et al. 1993; Marchand 1996; Demas and Nelson 1998; Boonstra et al. 2014; Varpe 2017), whereas winter, cold ambient temperature and food restriction are all connected to impaired immune function (Nelson and Demas 1996; Demas and Nelson 1998; Lochmiller and Deerenberg 2000; Cichoń et al. 2002; Huitu et al. 2007; Kusumoto 2009, Ksia̧zek and Konarzewski 2012). Hence, a chronic infection that is seemingly harmless during summer, could have more severe consequences for the individual during winter by consuming the already limited energy resources, with possibly a negative effect on body condition and even on survival (Kallio et al. 2007). In our experiment, this effect, however, was not observed.

The observed effect of food supplementation during winter on body condition in voles is supported by several earlier studies (Eccard and Ylönen 2001; Koskela et al. 2004; Ylönen & Eccard 2004; Von Blanckenhagen et al. 2007; Forbes et al. 2014; Johnsen et al. 2017). It is often recorded that under favourable conditions Arvicolinae species such as voles and lemmings may occasionally breed during winter, outside their breeding season (Eriksson 1984; Moffatt et al. 1993; Henttonen 2000; Sipari et al. 2016). Factors triggering winter breeding in voles are not well known, but abundant food sources seem to be one of the key factors (Eriksson 1984; Moffatt et al. 1993; Henttonen 2000; Forbes et al. 2014). In the study of Cayol et al. (Cayol et al. 2018), performed during the bank vole breeding season, *Borrelia* infection caused hastened reproduction in females, while impaired the breeding probability in large males. In our study, *Borrelia* infection did not affect the physiological signs of reproductive activity, however, the actual breeding was not monitored.

Due to drastically decreased survival later in the winter, our results only cover the period of late autumn/early winter. This limitation needs to be considered when interpreting the results. Furthermore, the artificial route of infection (via subcutaneous injection of bacteria suspension, rather than via tick bite) may result as a lower infectivity of *B. burgdorferi* sensu lato (Gern et al. 1993). Finally, introducing laboratory born animals into field conditions always bear the risk that some of the observed mortality is caused by the stress or their inability to thrive in the wild. However, it seems that the effects of a chronic *B. afzelii* infection are relatively negligible to its host even under winter conditions. The lack of observed differences in body mass, body condition index and in signs of breeding between infected and uninfected groups indicate that the energy resources in bank voles were not severely impaired by the infection during winter. These results suggest that the infection’s consequences on the host winter survival are likely insignificant, even though our results do not cover the whole winter.

The reasons why some of the endemic pathogens present in bank voles, such as *B. afzelii*, PUUV and cowpox virus all seem benign to the host during summer, but differ in their impact during winter are likely a result of complicated interactions between environment, host physiology and pathogen characteristics (Telfer et al. 2002; Kallio et al. 2007; Kloch et al. 2013). Ecological factors such as temperature, nutrition and reproductive status can affect not only the host but also the pathogen virulence, potentially leading to variable host–pathogen interactions between host populations under different environmental conditions (Blanford et al. 2002; Bajer et al. 2005; Mitchell et al. 2005; Scholthof 2007; Beldomenico & Begon 2010; Mills et al. 2010; Schroderus et al. 2015). This could partly explain the more notable effects of *Borrelia* infection in bank voles observed in summer (Cayol et al. 2018), compared to our results, suggesting no significant effects on the wintering host. Hence, our results support the idea of season dependent effects in host–pathogen interactions, a topic often neglected in disease ecological studies. Understanding the effect of seasonality and environmental conditions on pathogen–host interactions is essential when evaluating pathogen impact on host population dynamics, as well as for epidemiological models of zoonotic diseases. Limiting empirical disease ecological studies only to summer and breeding season will predispose the results to the risk of misinterpretation and erroneous extrapolations.

## Supplementary Information

Below is the link to the electronic supplementary material.

Supplementary file1 Online Resources 1: Supplementary tables 1 and 2. (PDF 729 KB)

Supplementary file2 Online Resources 2: Original data (XLSX 18 KB)

Supplementary file3 Online Resources 3: R codes used to analyse the data (PDF 307 KB)

## Data Availability

All data produced from this study are provided as electronic supplemental material (ESM), Online Resources 2.

## Code availability

All R codes used for the statistical analyses are provided as electronic supplemental material (ESM), Online Resources 3.
