# Supplementary material for: The effects of Borrelia infection on its wintering rodent host
Source: Oecologia. 2022 Oct 15;200(3-4):471–8. doi: 10.1007/s00442-022-05272-y (PMC9675652; doi:10.1007/s00442-022-05272-y)
Supplement: Supplementary file 1 — Supplementary file1 Online Resources 1: Supplementary tables 1 and 2. (PDF 729 KB) [file 442_2022_5272_MOESM1_ESM.pdf]

The effects of Borrelia infection on its wintering rodent host

Saana Sipari, Jukka Hytönen, Annukka Pietikäinen, Tapio Mappes, Eva R. Kallio

Electronic Supplemental Material (ESM)

Online Resources 1

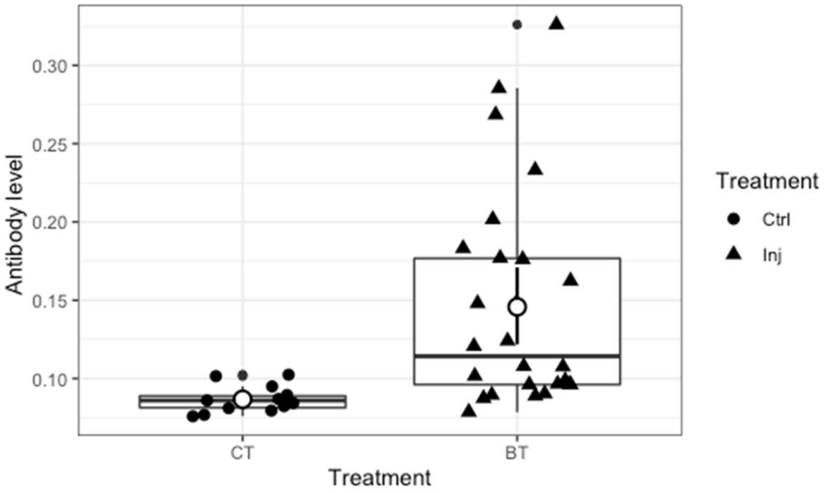

Figure S1. Antibody levels (mean absorbance) in relation to the original treatment (CT = Control individuals, BT= *Borrelia* injected individuals) in 40 bank voles captured and sampled in mid-December.

Table S1. The number of *Borrelia* injected (BT) and control (CT) individuals per enclosure (Enc) at the beginning of the experiment (Oct) and how many of them have been recaptured in mid-December (Dec). Also, the food treatment of each enclosure is provided. Only the original enclosure is provided although five individuals were found to have changed the enclosure inn December, and three of them changed from ‘No food’ to ‘Food’ treatment. These three individuals have been excluded from GLMM and LMM analyses (see Material and Methods; Statistical analyses)

Enc	BT Oct	BT Dec	CT Oct	CT Dec	Food treatment Food/No Food
1	2♀, 2♂	1♀, 1♂	2♀, 2♂	1♂	Food
2	2♀, 2♂	1♀	2♀, 2♂		No Food
3	2♀, 2♂	1♀, 2♂	2♀, 2♂	1♀	No Food
4	2♀, 2♂	1♀	2♀, 2♂		No Food
5	2♀, 2♂	2♀, 1♂	2♀, 2♂	2♀, 1♂	Food
6	2♀, 2♂	2♀, 2♂	2♀, 2♂	2♀, 2♂	No Food
7	2♀, 2♂	1♀, 1♂	2♀, 2♂	1♂	No Food
8	2♀, 3♂	2♂	2♀, 1♂	2♀, 1♂	Food
9	2♀, 2♂	1♀, 2♂	2♀, 2♂	1♂	No Food
10	2♀, 2♂	1♀, 2♂	2♀, 2♂	2♂	Food
11	2♀, 2♂	2♀, 1♂	2♀, 1♂	1♀, 1♂	No Food

Table S2. Model selection table for global models for a) bank vole survival October-December; b) Body mass in December; c) Body condition index in December; d) Antibody absorbance level in December. The global model shows all the variables that were included in the model selection procedure. Variables *Borrelia* treatment (Treatment), Food treatment (Food1), Sex and Initial body mass were included in all final models. * is a symbol for interaction that include all possible 2-way interactions and main effects or the variables. AB means antibody absorbance as explanatory variable. The simplest model within 2 AICc value of the lowest is the best model and marked with **bold**. All models within 2 AICc of the lowest AICc are provided. All models included a random effect *Enclosure in October.*

		df	AICc	ΔAICc
a) Survival till December				
Global model	# + Treatment + Food1 + Sex + Orig_mass + Treatment * Food1 * Sex + Treatment * Orig_mass * Sex			
	**# + Treatment + Food1 + Sex + Orig_mass**	**6**	**117.5**	-
	# + Treatment + Food1 + Sex + Orig_mass + Food1* Treatment	7	118.4	0.87
	# + Treatment + Food1 + Sex + Orig_mass + Food1* Sex	7	118.6	1.09
	# + Treatment + Food1 + Sex + Orig_mass + Orig_mass* Sex	7	119.3	1.81
b) Body mass in December				
Global model	# + Treatment + Food_1 + Sex + Orig_mass +Treatment * Food_1 * Sex + Treatment * Orig_mass * Sex			
	**# + Treatment + Food_1 + Sex + Orig_mass**	**7**	**188.3**	-
	# + Treatment + Food_1 + Sex + Orig_mass + Treatment* Sex	8	188.5	0.19
c) Body condition index in December				
Global model	# + Treatment + Food_1 + Sex + Orig_mass+ Treatment * Food_1 * Sex + Treatment * Orig_mass * Sex			
	**# + Treatment + Food_1 + Sex + Orig_mass**	**7**	**122.9**	-
	# + Treatment + Food_1 + Sex + Orig_mass + Food_1* Sex	8	123.6	0.77
d) Antibody absorbance levels in December				
Global model	# + Treatment + Food_1 + Sex + Orig_mass + Treatment * Food_1 * Sex			
	**# + Treatment + Food_1 + Sex + Orig_mass**	**7**	**-84.9**	-
