# Supplementary material for: The effects of Borrelia infection on its wintering rodent host
Source: Oecologia. 2022 Oct 15;200(3-4):471–8. doi: 10.1007/s00442-022-05272-y (PMC9675652; doi:10.1007/s00442-022-05272-y)
Supplement: Supplementary file 3 — Supplementary file3 Online Resources 3: R codes used to analyse the data (PDF 307 KB) [file 442_2022_5272_MOESM3_ESM.pdf]

The effects of Borrelia infection on its wintering rodent host

Saana Sipari, Jukka Hytönen, Annukka Pietikäinen, Tapio Mappes, Eva R. Kallio

Electronic Supplemental Material (ESM)

Online Resources 3

```
####Winter borrelia revision, with orig_mass, September 2022

setwd("C:/Users/saana/Documents/Borrelia_winter2020/Borreliawinter")

Data1<-read.csv("Data.csv")

#Excluding the three indiv, that changed from no-food to food treatment

Surv<-subset(Data1, Ind_in_analyses==1)

str(Surv)

library(MuMIn)
library(lme4)
library(nlme)

Surv_data <-subset(Surv,!is.na(Orig_mass))

#Global model for survival

M1<-
glmer(Survival~Orig_mass+Treatment+Food1+Sex+Treatment*Food1*Sex+Treatment*Orig_mass*Sex+(1|E
nc_Orig),family=binomial(link=logit),data= Surv_data,na.action="na.fail")

#Model comparison

summary(M1)
```

```
models1<-dredge(M1,subset=Food1&&Treatment&&Sex&&Orig_mass)

models1[1:15]


#Best model is


M2<-
glmer(Survival~Treatment+Food1+Sex+Orig_mass+(1|Enc_Orig),family=binomial(link=logit),data=Surv_dat
a,na.action="na.fail")

summary(M2)


#Body mass, global model for model comparison


Surv_data <-subset(Surv,!is.na(Bodymass))

Surv_data <-subset(Surv_data,!is.na(Orig_mass))


Mod1<-lme(Bodymass~Treatment + Food1 + Sex +Orig_mass+ Treatment * Food1 *
Sex+Treatment*Orig_mass*Sex,random=~1|Enc_Orig,method = "ML",data= Surv_data,na.action="na.fail")


summary(Mod1)

Models1<-dredge(Mod1, subset = Food1 && Treatment && Sex&&Orig_mass&&AB)

Models1[1:15]


#Best model for body mass


Mod2<-lme(Bodymass~Treatment + Food1 + Sex+Orig_mass+AB ,random=~1|Enc_Orig,method =
"REML",data=Surv_data,na.action="na.fail")

summary(Mod2)


#Body condition, global model and model selection
```

```
Surv_data <-subset(Surv,!is.na(Bodymass))

Surv_data <-subset(Surv_data,!is.na(Orig_mass)

summary(Surv_data)


condition<-lm(Bodymass~Head, data=Surv_data)

summary(condition)

plot(condition)


Surv_data$Cond<-rstandard(condition)


Mod3<lme(Cond~Treatment+Food1+Sex+Orig_mass+Treatment*Food1*Sex+Orig_mass*Treatment*Food1
,random=~1|Enc_Orig,method = "ML",data=Surv_data, na.action="na.fail")


summary(Mod3)

Models1<-dredge(Mod3, subset = Food1 && Treatment && Sex&&Orig_mass)

Models1[1:15]


#Best model for body condition index


Mod4<-lme(Cond~Treatment+Food1+Sex+Orig_mass,random=~1|Enc_Orig,method =
"REML",data=Surv_data, na.action="na.fail")


summary(Mod4)


#Breeding condition

##(Due to the low sample size and unevenly distributed cases between the treatment groups, no tests for
interactions are feasible)


Surv_data<-subset(Surv,!is.na(Orig_mass))

Surv_data <-subset(Surv_data,!is.na(Matu))
```

```r
M1<glmer(Matu~Food1+Sex+Treatment+Orig_mass+(1|Enc_Orig),family=binomial(link=logit),data=Surv_data,na.action="na.fail")

summary(M1)

#Antibodies, global model


Surv_data<-subset(Surv,!is.na(Orig_mass))

Surv_data <-subset(Surv_data,!is.na(AB))


str(Surv_data)


Mod1<lme(AB~Treatment+Food1+Sex+Orig_mass+Treatment*Food1*Sex+Orig_mass*Treatment*Food1,random=~1|Enc_Orig,method="ML",data=Surv_data, na.action="na.fail")


summary(Mod1)


Models1<-dredge(Mod1, subset = Food1 && Treatment&&Sex&&Orig_mass)

Models1[1:15]


#Best model for antibodies


Mod2<-lme(AB~Treatment+Food1+Sex+Orig_mass,random=~1|Enc_Orig,method="REML",data=Surv_data, na.action="na.fail")

summary(Mod2)


# Seroconversion figure


library(ggplot2)


BM_AB_data <-subset(Surv,!is.na(Bodymass))

BM_AB_data_red <-subset(BM_AB_data, !is.na(AB))


ggplot(BM_AB_data_red, aes(x=Treatment, y= AB))+
```

```
   geom_boxplot()+
  geom_jitter(width=0.2, size=2.5, aes(shape=Treatment))+
  #  geom_hline(yintercept = 31.03704, linetype= "dashed", color ="grey")+
  stat_summary(fun.data = mean_cl_boot,
             geom="pointrange",
             size=0.8 ,
             shape=21,
             fill="white")+
  #scale_y_continuous(NIP = seq(0, 0.6, by=0.10))+
   scale_x_discrete(labels=c("CT", "BT"))+
  xlab("Treatment")+
  ylab("Antibody level ")+

  #scale_color_manual(values = c("yellow", "blue"),
  #                  name=c("Wool Types"),
  #                  labels=c("Llhama", "Acrylic"))+
  #coord_flip()+
  theme_bw()



#Seroconversion test, Fisher's


dat <- data.frame(
  "Pos" = c(13, 0),
  "Neg" = c(14, 13),
  row.names = c("Cont", "Inj"),
  stringsAsFactors = FALSE
)
colnames(dat) <- c("Pos", "Neg")
dat
```

```
test <- fisher.test(dat)

test

test$p.value

# Are there differences in the initial body mass between groups (are individuals randomly distributed into groups)?


# all indiv. included


Groups_data <-subset(Data1,!is.na(Orig_mass))

t.test(Orig_mass~Treatment,data=Groups_data)
```
